# Using a multiscale image processing method to characterize the periodic growth patterns on scallop shells

**DOI:** 10.1002/ece3.2789

**Published:** 2017-02-09

**Authors:** Qiang Xing, Tengda Wei, Zhihui Chen, Yangfan Wang, Yuan Lu, Shi Wang, Lingling Zhang, Zhenmin Bao

**Affiliations:** ^1^ Key Laboratory of Marine Genetics and Breeding (Ministry of Education)College of Marine Life SciencesOcean University of ChinaQingdaoShandongChina; ^2^ School of Mathematical SciencesOcean University of ChinaQingdaoShandongChina; ^3^ College of Life ScienceUniversity of DundeeDundeeUK; ^4^ College of Information and EngineeringOcean University of ChinaQingdaoShandongChina; ^5^ Laboratory for Marine Fisheries Science and Food Production ProcessesQingdao National Laboratory for Marine Science and TechnologyQingdaoChina; ^6^ Laboratory for Marine Biology and BiotechnologyQingdao National Laboratory for Marine Science and TechnologyQingdaoChina

**Keywords:** growth patterns, individual recognition, MATLAB package, multiscale image processing, scallop shell, space‐based depth‐first search algorithm

## Abstract

The fine periodic growth patterns on shell surfaces have been widely used for studies in the ecology and evolution of scallops. Modern X‐ray CT scanners and digital cameras can provide high‐resolution image data that contain abundant information such as the shell formation rate, ontogenetic age, and life span of shellfish organisms. We introduced a novel multiscale image processing method based on matched filters with Gaussian kernels and partial differential equation (PDE) multiscale hierarchical decomposition to segment the small tubular and periodic structures in scallop shell images. The periodic patterns of structures (consisting of bifurcation points, crossover points of the rings and ribs, and the connected lines) could be found by our Space‐based Depth‐First Search (SDFS) algorithm. We created a MATLAB package to implement our method of periodic pattern extraction and pattern matching on the CT and digital scallop images available in this study. The results confirmed the hypothesis that the shell cyclic structure patterns encompass genetically specific information that can be used as an effective invariable biomarker for biological individual recognition. The package is available with a quick‐start guide and includes three examples: http://mgb.ouc.edu.cn/novegene/html/code.php.

## 1 Introduction

Mollusk shells are composed of mainly calcium carbonate (95% by mass) and other inorganic substances (0.01%–5% by mass), both of which are secreted by the shellfish mantle that encloses, supports, and protects its soft part (Weiner & Lowenstam, 1989). Due to seasonal changes in the physical and chemical properties of seawater in the environment, the rates of secretion and deposition of shell materials are varied, resulting in a periodic appearance of growth patterns on shells with different densities in the internal structure and chemical elements (Michio & Hiromichi, 2013). Using a combination of mark–recapture methods, the features of these rings in mollusk shells can be adapted and applied to studies on the population biology of the scallop (Allison & Brand, 1995; Berkman, 1990), which are used to determine the habitat value of the ecosystems for fishery restoration and for enhancement through stocking.

Perhaps the most important tool of population biology is the ability to recognize and track individual animals over space and time. Traditionally, this recognition has been accomplished by capturing animals and placing visible and unique marks on them (Williams, Nichols, & Conroy, 2002). Recently, molecular genetic markers such as RFLP (restriction fragment length polymorphism), RAPD (random amplified polymorphism DNA), SSR (simple sequence repeat), and SNP (single nucleotide polymorphism) have also been widely used to study the population and individual recognition (Reed, Tollit, Thompson, & Amos, 1997; Wang, 2015). However, these methods are not suitable to a larger population because of inconsistency, inconvenience and higher cost, among others. Currently, photographic mark–recapture (PMR) has gained popularity because of the advances in digital photography and image processing software. The abundance of species with variable natural marking patterns makes this an attractive method for many researchers. PMR has been employed particularly in the studies of populations of marine mammals and mammalian terrestrial predators (Fearnbach, Durban, Parsons, & Claridge, 2012; Forcada & Aguilar, 2000; Karanth & Nichols, 1998; Langtimm et al., 2004). Some image analysis algorithms have been used to extract, store, and compare pattern information from digital images (Bolger, Morrison, Vance, Lee, & Farid, 2012).

In this study, the growth patterns on the shells of Yesso scallop (*Patinopecten yessoensis*), Weathervane scallop (*Patinopecten caurinus*), and Zhikong scallop (*Chlamys farreri*) were analyzed using a multiscale image processing method to collect the periodic structures of growth patterns on shells for scallop individual recognition. First, we used a Gaussian‐matched filter to fit the growth patterns into enhanced lines. Next, we applied a total variation model (Rudin, Osher, & Fatemi, 1992), which was particularly effective with the enhanced image for line localization. The multiscale line maps generated by multiscale hierarchical decomposition contained a series of segmentation results at varying image resolutions of shell patterns details at different levels. This process performed an iterative segmentation at an increasing image resolution in each step, and thus detected much smaller growth rings and ribs. Finally, the periodic structures, consisting of bifurcation points, crossover points of the rings and ribs, and the connected lines, could be acquired by our Space‐based Depth‐First Search (SDFS) algorithm. Meanwhile, we created a MATLAB package to implement our method for segmentation and cyclic pattern extraction of the growth rings on the scallop shell CT and digital images available in this study. The feasibility, effectiveness, and accuracy of our method were also assessed in identifying group sizes of the three species in a mixed population.

## 2 Materials and Methods

### 2.1 Sampling selection for scallop

One hundred of each species, the two‐year‐old Yesso scallop, Weathervane scallop, and the Zhikong scallop, were collected from natural populations at Dalian Zhangzidao Fishery Group Co. (Liaoning Province, China) in October 2016. Immediately after collection and sorting, scallop shells were obtained through removing encrusters from the shells, and washed three times by distilled water and then dried for later use. CT images were acquired by a 200‐ma Medical X‐ray Digital Equipment (KH200‐I), and the camera images were taken by a Nikon D800 camera.

### 2.2 Multiscale segmentation

The segmentation of the growth rings is the most important procedure in the identification of scallop shell rings and ribs. For the rings and ribs detection, we used matched filtering with Gaussian kernel (MFGK) $\text{ker}\;\left(x,\;y;a,\;b\right)=-\text{exp}\left(\frac{-{\left(a^{-1}\left(x-b\right)\right)}^{2}}{2\sigma^{2}}\right)$, and the computed MFGK response image was as follows:

(1) $$M_{\ker}\left(x,\;y;\;a,\;b\right)={\text{max}}_{\theta }\left(r_{\theta }\left(\text{ker}\left(x,\;y;\;a,\;b\right)\right)*\text{Img}\left(x,\;y\right)\right),$$

where Img, (*x*,* y*), ker, *a*, and *b* denoted a shell image, a two‐dimensional pixel position, two‐dimensional Gaussian functions (Chaudhuri, Chatterjee, Katz, Nelson, & Goldbaum, 1989), the dilation parameter (also known as scaling parameter), and the translation parameter, respectively. *r*_θ_ rotated the kernel function with an angle θ, and * represented the convolution operation in variables (*x* and *y*).

**Figure 1:**
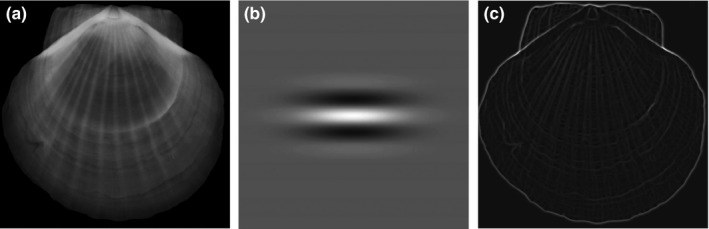
Enhancement of CT image: (a) CT image of scallop; (b) two‐dimensional Gaussian kernel; (c) enhanced image by matched filtering with Gaussian kernel (MFGK)

To illustrate, Figure 1 shows the MFGK response *M*_ker_(*x*,* y*;* a*,* b*) in Equation (1), which was calculated with a number of discrete values of θ from 10° up to 170° with an increment of 10° and *a* = 8. As shown in Figure 1, the small rings and ribs in the original image were enhanced by MFGK.

The normalized response image was defined as follows:

(2) $$f=\frac{M_{\ker}-\mu }{\sigma }$$

where μ and σ were the mean and standard deviation of the enhanced MFGK image *M*_ker_(*x*,* y*;* a*,* b*).

The multiscale hierarchical decomposition of an image *f* was defined as follows (Wang, Ji, Lin, & Trucco, 2013). Given an initial scale parameter λ_0_ and the total variation (TV) function (Rudin et al., 1992) $J\left(f,\lambda \right)=\lambda ||v_{\lambda }{\left|\right|}_{L^{2}}^{2}+\left|\right|u_{\lambda }{\left|\right|}_{\mathrm{BV}}$, BV stood for the homogenous bounded total variation space equipped with the norm of total variation

(3) $$\begin{array}{cc} & {\left||.|\right|}_{\mathrm{BV}}={\left||\nabla |\right|}_{L^{1}}=\int_{\Omega }\sqrt{{\left(u_{\lambda }\right)}_{x}^{2}+{\left(u_{\lambda }\right)}_{y}^{2}}\\ & f=u_{0}+v_{0}\;\text{where}\;\left[u_{0},\;v_{0}\right]{:=\text{argmin}|}_{u+v\;=\;f}J\left(f,\;\lambda_{0}\right);\\ & v_{k}=u_{k+1}+v_{k+1},\;k=0,1,\ldots ,\\ & \text{where}\;\left[u_{k+1},\;v_{k+1}\right]{:=\text{argmin}|}_{u+v\;=\;v_{k}}J\left(v_{k},\;\lambda_{0}2^{k+1}\right). \end{array}$$

Based on the above enhancement with MFGK and multiscale hierarchical decomposition, many line maps *u*_*k*_ were generated at varying image resolutions, representing different levels of line details to avoid the possible failure of feature extraction caused by a single‐scale segmentation.

The multiscale segmentation results were further processed by the following procedures for better feature extraction: (1) removed the connected regions with few pixels for denoising and (2) filled the line holes based on erosion and dilation method (Gonzalez & Woods, 2007).

**Figure 2:**
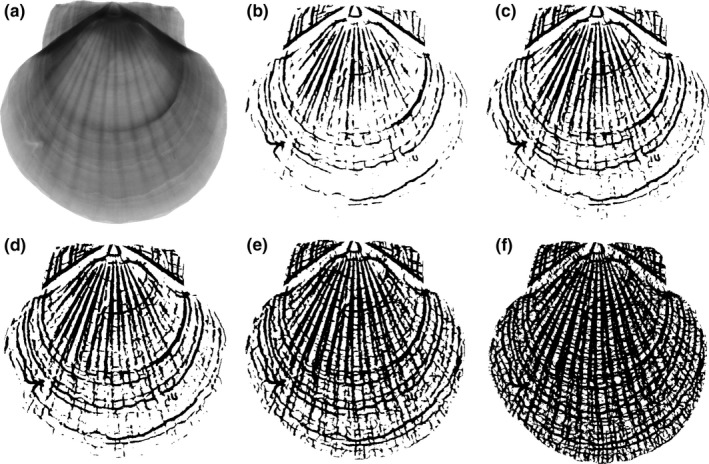
Segmentation results produced by multiscale hierarchical decomposition with λ_0_ = 0.01 and λ_*i*_ = λ_0_2^*i*^: (a) original CT image; (b–f) segmented image at scaling parameters λ_1_, λ_2_, λ_3_, λ_4_, and λ_5_, respectively

Figure 2 presents illustrative segmentation results for X‐ray images of a scallop. The initial scaling parameter was λ_0_ = 0.01 in the multiscale hierarchical decomposition.

### 2.3 Multiscale cyclic structures

To get a more accurate pattern matching, multiscale cyclic structures were extracted from the multiscale segmentation results to compute similarity between two pairs of images by our method.

#### 2.3.1 Cyclic structure extraction

The extraction procedure of cyclic structures could be achieved by the following four steps:

##### 2.3.1.1 Skeletonization

The binary multiscale line networks segmented in the previous step were skeletonized to identify the two‐dimensional centerlines of individual branches and to determine the branch point locations of the cycle structures. A skeletonization algorithm based on morphological operations (Gonzalez & Woods, 2007) was customized for our application.

##### 2.3.1.2 Detection of feature points

After the skeletonization step, the feature points, which consisted of the bifurcation and crossover points in the binary skeleton line networks, were searched as follows. First, the pixels where the value sum of the 8‐adjacent pixels was greater than 2 in connected components were labeled; the pixels could not be bifurcation or crossover points if the sum of its 8‐adjacent pixels was less than or equal to 2. Next, the center points of all the connected components were detected as candidate feature points, and they were considered vertices (nodes), while their connected lines were considered edges (links) in graph theory. Last, the feature points were determined by removing the candidate vertices whose degree was equal to 1 iteratively, as a vertex belongs to a cycle only if it had at least two connected vertices.

##### 2.3.1.3 Acquisition of cyclic structures

Finding the cyclic structures from the segmented network was performed by computing the minimum cycle basis from the undirected and unweighted graph (Mehlhorn & Michail, 2009). However, because the feature points have different fixed spatial positions in the image, the spatial information of the feature points and the cross‐product of the vectors that form these points were used to develop the Space‐based Depth‐First Search (SDFS) algorithm (Algorithm 1) to find the cyclic structures.

##### 2.3.1.4 Description and matching of cyclic structures

**Figure 3:**
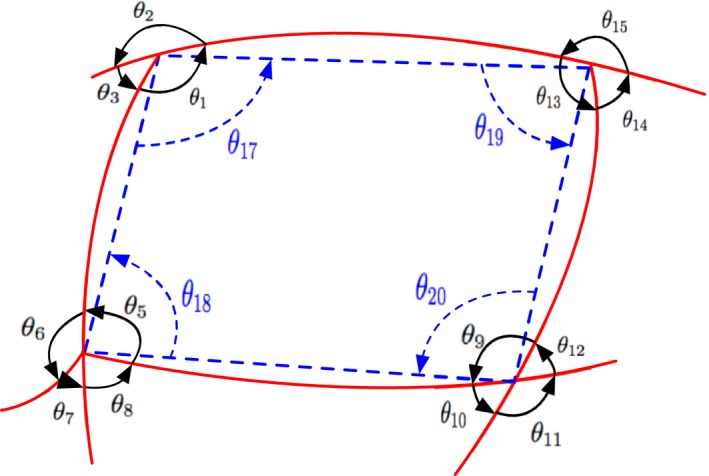
Cyclic structure description: $L_{1}∼L_{4}$, θ_1_ ~ θ_16_, and θ_17_ ~ θ_20_ represent branch lengths, branch angles, and angles between adjacent edges, respectively, in a four‐point cyclic structure

To make the structure more unique, we described a cyclic structure using branch lengths, branch angles, and angles between adjacent edges, and Figure 3 shows an example description of a four‐point cyclic structure. The lengths were calculated based on the pixel distance, and the angles were calculated relying on the adjacent points in the radial lines or edges. Then, we normalized all the lengths and angles using Equations (4) and (5) to preserve the scaling invariant and the guarantee rotation invariant, respectively, yielding the final feature vector given in Equation (6) as an example.

(4) $$L_{i\mathrm{Norm}}=\frac{L_{i}}{\sum L_{i}}$$

(5) $$\theta_{j\mathrm{Norm}}=\frac{\theta_{j}}{360^{\circ }}$$

(6) $$\begin{array}{cc} \tilde{v}=\left\{\text{lengths, angles}\right\}= & \{L_{1},L_{2},L_{3},L_{4},\theta_{1},\theta_{2},\theta_{3},0,\theta_{5},\theta_{6},\theta_{7},\theta_{8},\theta_{9},\theta_{10},\\ & \theta_{11},\theta_{12},\theta_{13},\theta_{14},\theta_{15},0,\theta_{17},\theta_{18},\theta_{19},\theta_{20}\} \end{array}$$

The number of feature vector elements varies with the number of cyclic feature points, and the number of branch angles varies with the style of feature points; for example, a bifurcation point usually had three branch angles, while a crossover point had four branch angles. To obtain consistent vector length, we considered the feature points of the cycle structure as all bifurcation or crossover points, making a maximum four branch angles for each point, and the feature vector of three‐, four‐, five‐, and six‐point cyclic structures became 18, 24, 30, and 36 dimensions (the missing elements were set to 0 as demonstrated in Equation (6)), respectively.

The feature matching process can search for good similarity among all structure pairs. Matching two cyclic structures with the same number of feature points could be implemented by minimizing the similarity measure:

(7) $$s_{ij}=d\left({\tilde{v}}^{i},{\tilde{v}}^{j}\right)$$

where ${\tilde{v}}^{i}$ and ${\tilde{v}}^{j}$ denoted the characteristic vectors of *i*th and *j*th cycle structure in two images. The term “*d*(.)” denoted the measured distance between the characteristic vectors.

**Figure 4:**
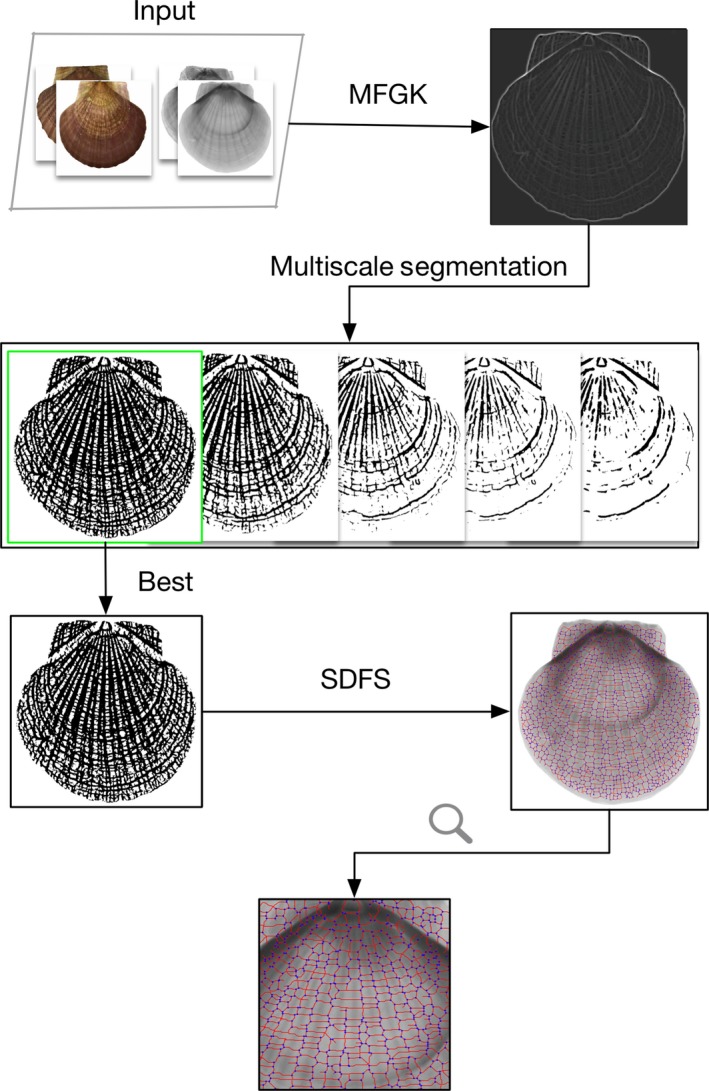
Flowchart of our multiscale PDE‐based method for segmentation and identification of scallop shell growth rings

The essential architecture of our method for fully automated segmentation and identification of shell growth rings is shown in Figure 4.

## 3 Results

### 3.1 Segmentation

**Figure 5:**
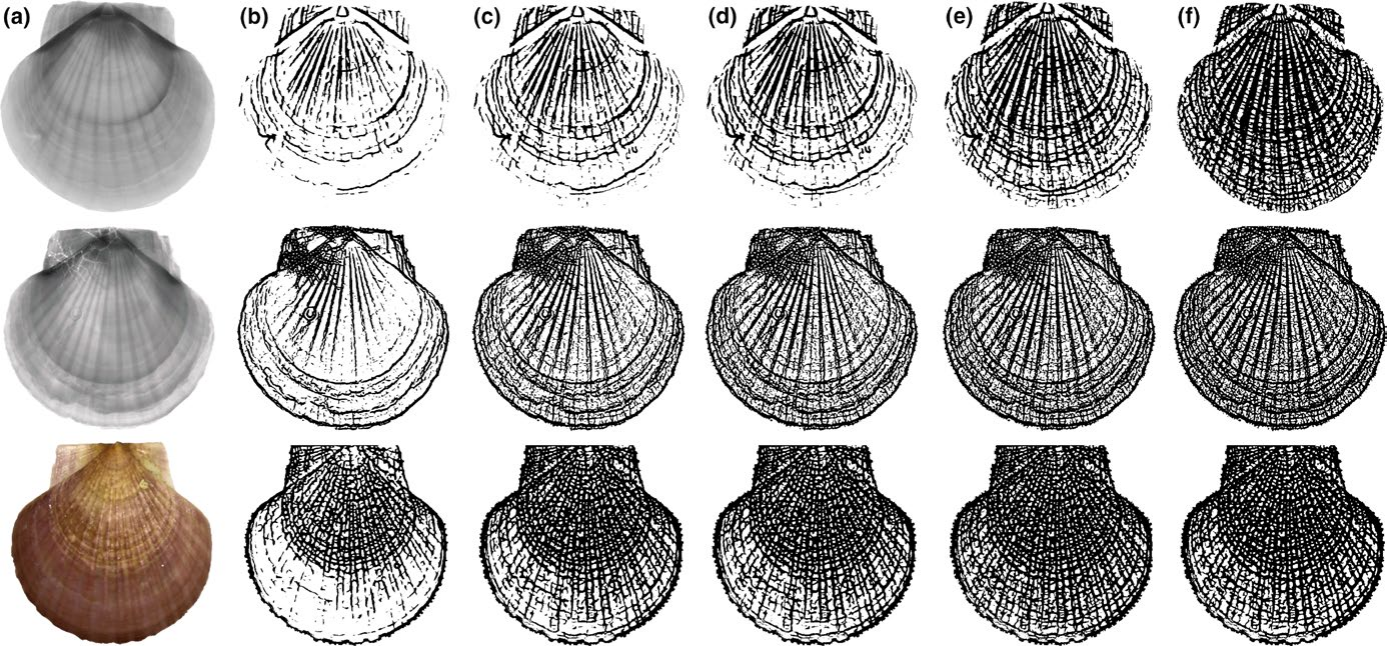
Comparisons of segmentation results by our method at different scaling parameters: (a) original images; (b–f) segmentation results by our method at different scaling parameters.

The results of illustrative segmentation using our multiscale PDE‐based method with different scale parameters are shown in Figure 5, including CT images (top and middle) and a camera image (bottom). Obviously, CT images (top and middle) and camera images can be used for good segmentation with the selection of more branch points and growth rings and ribs. Meanwhile, the segmentation of growth rings in the camera image can be still detected even though the original image was degraded by some shell color; hence, our segmentation method was robust in noise and color.

### 3.2 Comparison with other segmentation methods

**Figure 6:**
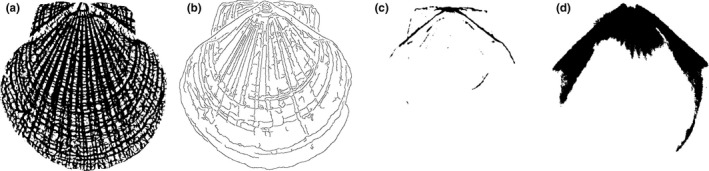
Comparisons of segmentation results using different methods: (a) our method; (b) edges detected by the Canny algorithm; (c) morphology method; (d) histogram thresholding method

To verify the segmentation performance of our method on the network of growth rings, we compared the present experiment results with the traditional method of image segmentation, including the Canny algorithm, morphology method, and histogram thresholding method (Gonzalez & Woods, 2007). As shown in Figure 6, our method produced the best segmentation on shell individual rings and ribs and key growing points.

### 3.3 Identification of radial ribs and growth rings

**Figure 7:**
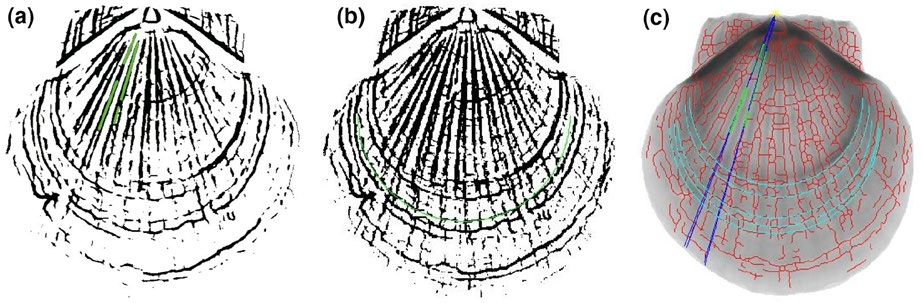
Identification of radial ribs and growth rings: (a) Radial ribs were found by the Hough transform for line detection and are shown in green; (b) growth rings were found by the neighborhood‐suppression method and are shown in green; (c) radial ribs crossed at the original growth point and crossed with growth rings

The processes to find radial ribs and growth rings are described as follows. First, the segmented image with a scale was divided into six sections every 30°. In each part, a few radial ribs on the segmented image were found by the Hough transform (Gonzalez & Woods, 2007) for line detection with an angle from 0° to 30°. The radial ribs on every part were extended and crossed at the original growth point as shown in Figure 7a. Second, we use the neighborhood‐suppression method (Kovács & Julesz, 1993) for peak finding to ensure that we found spatially separated circles for the detection of growth rings as shown in Figure 7b. Lastly, the distance of radial ribs between two consecutive growth rings, as shown in Figure 7c, was used to calculate the growth rate.

### 3.4 Identification of the growth patterns

**Figure 8:**
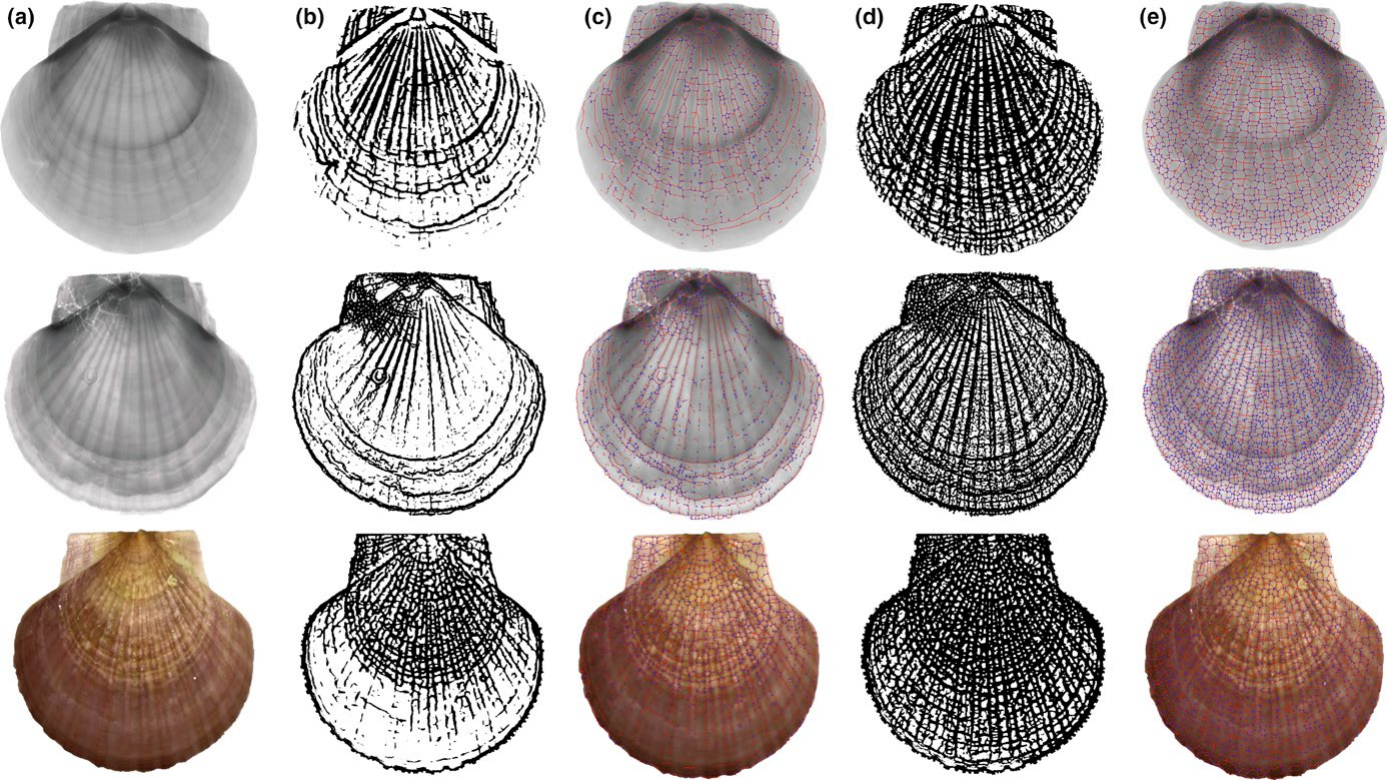
Cyclic structures of the Yesso scallop: (a) original images; (b) segmentation results with large scale; (c) identification results of cyclic structures for (b); (d) segmentation results with small scale; (e) identification results of cyclic structures for (d)

Using our SDFS algorithm, the cyclic structures consisting of bifurcation points, crossover points of the growth rings and ribs, and the connected lines were identified. Figure 8 presents multiscale segmentation and identification results of two CT images and a camera image with different scale parameters. Obviously, the more exquisitely the growth rings and ribs were segmented, the more delicate cyclic structures were presented.

**Figure 9:**
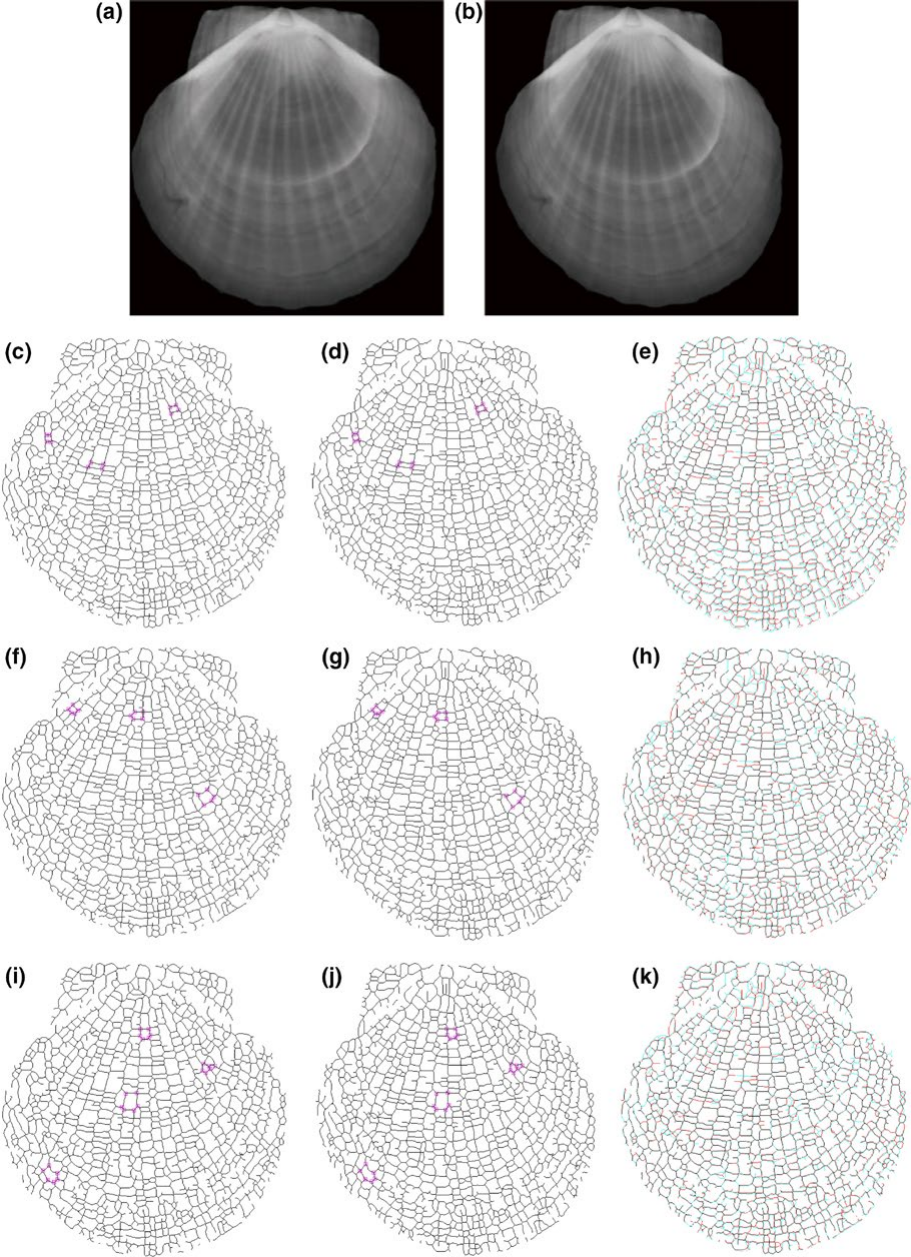
Image registration results of skeletons from multiscale segmentation results at multiscaling parameter λ_4_: (a) one scallop CT image; (b) another scallop image captured at different time; (c) skeleton and matched four‐point cyclic structures of (a); (d) skeleton and matched four‐point cyclic structures of (b); (e) the mosaic image with four‐point cyclic structures; (f) skeleton and matched five‐point cyclic structures of (a); (g) skeleton and matched five‐point cyclic structures of (b); (h) the mosaic image with five‐point cyclic structures; (i) skeleton and matched six‐point cyclic structures of (a); (j) skeleton and matched six‐point cyclic structures of (b); (k) the mosaic image with six‐point cyclic structures

Meanwhile, registration was adopted to test our SDFS algorithm using pattern matching in the cyclic structures of skeletons from multiscale segmentation results. Figure 9 presents the image registration results for multiscale segmentations at multiscaling parameters λ_4_. Figure 9a,b depicts a pair of CT scallop images taken at different times. The best matching four‐point, five‐point, and six‐point cycle structures are highlighted in Figure 9c,d,f,g,i,h, respectively, using square boxes. We tried linear, affine, and quadratic transformation and found that the affine model was enough and robust to describe the transformation. The mosaic images aligned with the affine models of four‐point, five‐point, and six‐point cyclic structures are shown in Figure 9e,h,k, respectively.

### 3.5 Individual recognition

For each species, 50 scallops of Yesso scallop, Weathervane scallop, and Zhikong scallop were randomly selected for capturing images. Each individual scallop had pictures taken twice in a 30‐day interval, and each time, 40 segmented images were captured, and thus, 80 segmented images were produced in total for an individual scallop. Next, we divided them into two halves, with one half receiving 40 images used for training and the remaining half used for testing. Meanwhile, we randomly selected an image as a template image for the training images for every individual scallop.

#### 3.5.1 Feature extraction

**Figure 10:**
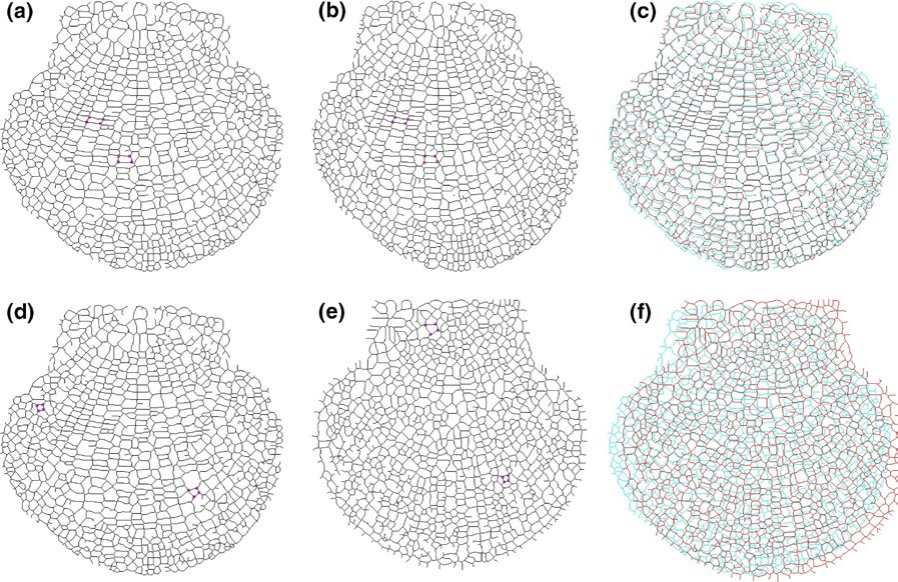
Overlap results: (a) skeleton and matched four‐point cyclic structures of individual images; (b) skeleton and matched four‐point cyclic structures of the same‐individual images captured at a different time; (c) the overlap image after registration for the same individual; (d) skeleton and matched four‐point cyclic structures of (a); (e) skeleton and matched four‐point cyclic structures of another individual image; (f) the overlap image after registration for the different individual

The feature set should be selected such that the between‐class discrimination is maximized, while the within‐class discrimination is minimized. Indeed, in order to avoid the curse of dimensionality, it is desirable for the feature set to be as small as possible. We defined the minimum value of the similarity measure as ms = min{*s*_*ij*_} and used ms as a feature for individual identity. Considering that there might exist some similar cyclic structures in different‐individual segmented images, an overlap ratio after registration was selected as another feature for individual identity. Figure 10 presents overlap results after same‐individual and different‐individual registration. It was observed that most of the rings aligned well, except for a few local centerlines with a 1‐pixel shift for same‐individual registration, and the overlap ratio, defined as ra, was over 50 percent in (c). Very few rings were aligned after different‐individual registration in (f), even when there were very similar cyclic structures in (d) and (e). For every individual, the corresponding feature classes were defined as C1 {same‐individual image features}, obtained by calculating {ms, ra} with its template image and the other 39 same‐individual training images, and C2{different‐individual image features}, obtained by computing {ms, ra} with its template image and the other 149 different‐individual training template images.

#### 3.5.2 Supervised classification

We used a neural network (NN) with three layers to obtain individual recognition. The number of inputs was equal to the dimension of the feature set. The output nodes were set at 2 so that the NN could classify into one of the same‐individual or different‐individual feature classes. The hidden layer nodes were 10. To maintain a certain level of confidence in the results, NN classification experiments were repeated 10 times. Training was conducted until the average error fell below 0.01 or reached a maximum iteration limit of 500. The average error denoted the error limit to stop NN training. The average error was the average of NN target output subtracted from the desired target output from all the training patterns. The desired target outputs were set to [1 0] for the class representing the same individual, while for the rest of the feature classes, it was set to [0 1].

The average results for individual recognition using four‐, five‐, and six‐point cyclic structures from multiscale segmentation results are given in Table 1. In general, the high averaged classification performance >99.10% validates the ability of the proposed method to identify individuals. The average classification performance >99.10% means that approximately 5,946 of 6,000 testing images were correctly classified into their corresponding individual classes. Obviously, the more delicate cyclic structures were identified; the higher accuracy was obtained using our multiscale segmentation method. The process of feature generation was basically the calculation of the similarity measures of cyclic structures in the segmented images and overlap ratio after registration. Because the dimension of the feature was fixed, the classification of feature vectors was fast. On a machine with an Intel Core i5‐3230 M CPU processor @2.60 GHz with 4 GB of RAM memory, the feature generation for training 150 individual samples took up to 3 hr, while the classification of the individual images took less than 20 s.

**Table 1:** Averaged classification performance

Scale parameters	Classification accuracy (%)				
	λ_1_	λ_2_	λ_3_	λ_4_	λ_5_
Four‐point cyclic structures	99.12	99.34	99.69	99.93	99.95
Five‐point cyclic structures	99.04	99.42	99.68	99.94	99.98
Six‐point cyclic structures	99.25	99.61	99.79	99.97	99.99
Average rate	99.13	99.46	99.72	99.94	99.97

#### 3.5.3 Species identification

**Figure 11:**
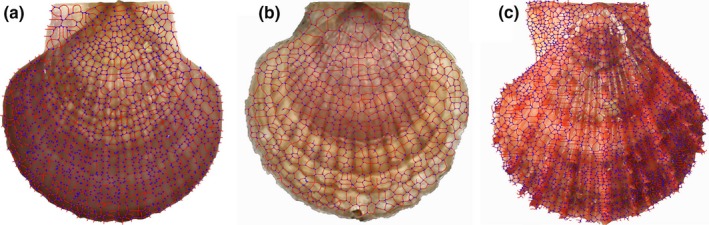
Shapes of the Yesso scallop, Weathervane scallop, and Zhikong scallop: (a) identification results of cyclic structures for a Yesso scallop; (b) identification results of cyclic structures for a Weathervane scallop; (c) identification results of cyclic structures for a Zhikong scallop

**Figure 12:**
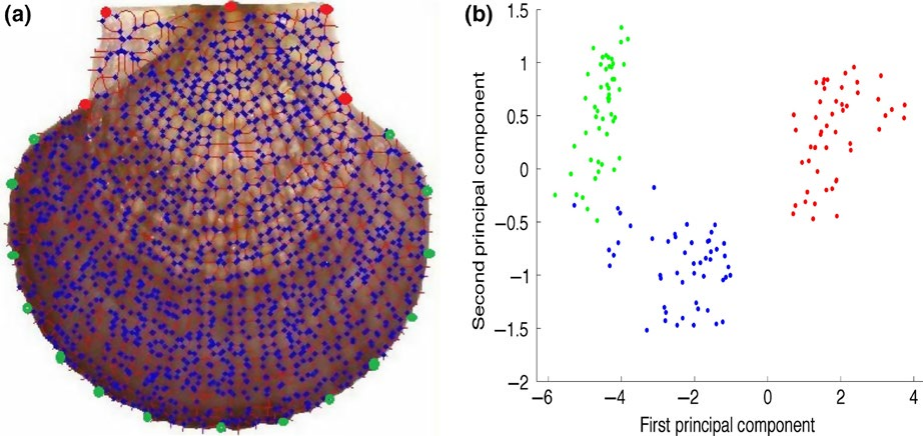
(a) The surface of a scallop, with the positions of landmarks and semilandmarks indicated. Fixed landmarks are shown as red points, semilandmarks along the edge of the shell are shown as green points, and surface semilandmarks on the scallop valve are shown as blue points. (b) Principal components plot of shell shape variation for the individuals used in this study: The first two principal component (PC) axes explained 81.2% of the total variation in shell shape (PC1 = 67.1%; PC2 = 14.1%). Species are designated as follows: Yesso scallop (green points), Weathervane scallop (blue points), and Zhikong scallop (red points)

We further tested the effectiveness of the above individual recognition method in identifying the three species Yesso scallop, Weathervane scallop, and Zhikong scallop, which are generally indistinguishable from each other by using the naked eye. Fifty scallops of each species were analyzed. As shown in Figure 11, the shapes of Yesso and Weathervane scallops, which belong to the same genus, were similar in shell shape and difficult to differentiate. Although the shape of Zhikong scallop was a little different compared to the Yesso scallop and Weathervane scallop, it was still a challenge to identify it in a big population of the three mixed species by manual sorting. Thus, we used the landmark‐based geometric morphometric method (Adams, Rohlf, & Slice, 2004; Bookstein, 1993) to quantify overall shell shape for precise sorting. An important advantage of this approach is that shape information from homologous anatomical structures (landmarks), and points along curves and points on anatomical surfaces could be included in the same analysis and were termed semilandmarks. In this paper, we first obtained high‐resolution bifurcation or crossover points of the shell image of each individual using our cyclic structures identification method. We then digitized the locations of five homologous landmarks on each segmented image, which are shown as red points in Figure 12a. Next, we digitized 15 semilandmarks along the ventral edge of the shell. Finally, we quantified the general shell surface by digitizing 100 cyclic structures as semilandmarks on the surface texture of each image. To accomplish this, we selected 100 cyclic structures from relatively evenly spaced surface on a single shell and treated this as a template. We then used the thin‐plate spline to warp the template to a second shell, using the fixed and edge landmarks as points of correspondence between the template and the shell. The cyclic structures on the image nearest to the 100 cyclic structures on the template were then taken as the surface semilandmarks for that individual. This was repeated on all individuals to obtain surface semilandmarks. We utilized this procedure to capture the general shape of the shell surface because the number of ridges per shell is not consistent among species or among individuals within a species. Once all individuals were digitized, we aligned them using a generalized Procrustes superimposition (Rohlf, 1965). From the aligned shells, a set of Procrustes shape coordinates were obtained. For the pair of species, we calculated the difference in average shell shape as the Euclidean distance between species means using Procrustes shape coordinates. We also performed a principal components analysis (PCA) to visualize the patterns of variation within and among the species. As shown in Figure 12b, the first principal component axis (PC1) and the second principal component axis (PC2) described 67.1% and 11.4% of the total variation, respectively. The two species between the Yesso scallop and Zhikong scallop or between the Weathervane scallop and Zhikong scallop could be sorted on the basis of shell shape by PC1 alone; however, there was also significant sorting between species when viewed by both PC1 and PC2. Importantly, the same genus species Yesso scallop and Weathervane scallop overlapped considerably in the morphospace, but 96% of individuals could be correctly sorted into their corresponding species using our method.

## 4 Discussion

The approach described in this paper using image processing analytical methods, which are widely used in studies on ecology and evolution (Bolger et al., 2012), has demonstrated its powerful application in studies on the growth patterns on bivalve shells. The patterns of periodic structures are composed of bifurcation points, crossover points of the growth rings and ribs, and the connected lines. The characteristic vector of each bifurcation structure consists of normalized branching angle and length, which is invariant against translation, rotation, scaling, and even modest distortion. This can greatly reduce mismatching during the matching process. As long as the cyclic pattern can be extracted from each shell, the individuals can be effectively identified by using the BP neural network (BPNN) classification of features of four‐, five‐, and six‐point cyclic structures from multiscale segmentation. The results showed that through the improvement in the extraction of cyclic structures and BPNN training, the accuracy for individual recognition could achieve nearly 100% for all subjects examined.

This individual recognition method can be easily applied to species identification. Species identification is necessary because Yesso scallop, Weathervane scallop, and Zhikong scallop, as major economic aquaculture species, form mixed species in many areas around the China. Yesso scallop and Weathervane scallop, as cold‐water species, prefer food‐rich environment and have relatively poor ability to resist pollution (Shumway & Parsons, 2011). In contrast, the Asian‐Pacific species, Zhikong scallop, performs better in warm and low‐food availability water (Guo & Luo, 2006). One of the most compelling patterns observed in evolutionary biology is that of morphological and behavioral convergence among species inhabiting similar environments. Using our method, the Yesso scallop and Weathervane scallop, which inhabit similar environments and were more similar in their shell shape, could be easily sorted, which will be useful for scallop machine sorting in the future.

Our method can also save time and cost less in comparison with molecular methods for the individual identification of scallops, especially in a large population. For 100 individual samples, it would take approximately 14 days for good identification with the traditional molecular methods (Wang, 2015; Wang, Meyer, Mckay, & Matz, 2012). In addition, these methods are generally laborious and time‐consuming and sometimes require invasive operations that need a relatively large amount of sample materials, which would require the sacrifice of animals under study to ensure a sufficient amount of DNA for individual recognition (Mao et al., 2013). However, our method can achieve a high‐throughput operation with aid of a CT machine, an ordinary digital camera, and even mobile phones and can reduce the workload to just less than 3 hrs. Therefore, we would propose that the periodic structures of scallop could be adapted to photographic mark–recapture (PMR) to study a large population of scallops.

Genetic breeding for a higher growth rate is one of the main focuses of the scallop farming industry (Gjedrem, 1983). There are many ways to determine the growth rates in mollusk aquaculture (Michellej & Amandae, 2008). Our method of scallop individual recognition could be used to calculate the growth rate easily by characterizing shell images during a time of scallop growth and development, thus allowing the subsequent study of these growth rates as they are influenced by environmental factors or aquaculture techniques. Indeed, the establishment of a method with a possible function in predicting the growth rate in relation to scallop developmental traits could provide useful information for targeting genetic improvement of this species.

## Conflict of Interest

All authors, Q.X., T.W., Z.C., Y.W., Y.L., S.W., L.Z., and Z.B., declare that they have no conflict of interest.

## Author Contributions

Z.B., Y.W., and Z.C. conceived and designed the study. Q.X and T.W were involved in preparation of shells for image processing and conducted the major part of MATLAB package for data analysis. Z.B., Y.W., Z.C., Y.L., S.W., and L.Z. drafted the manuscript. All authors read and approved the final manuscript.

## Ethical Approval

All applicable international, national, and/or institutional guidelines for the care and use of animals were followed.

## Algorithm 1

### Space‐based Depth‐First Search (SDFS)

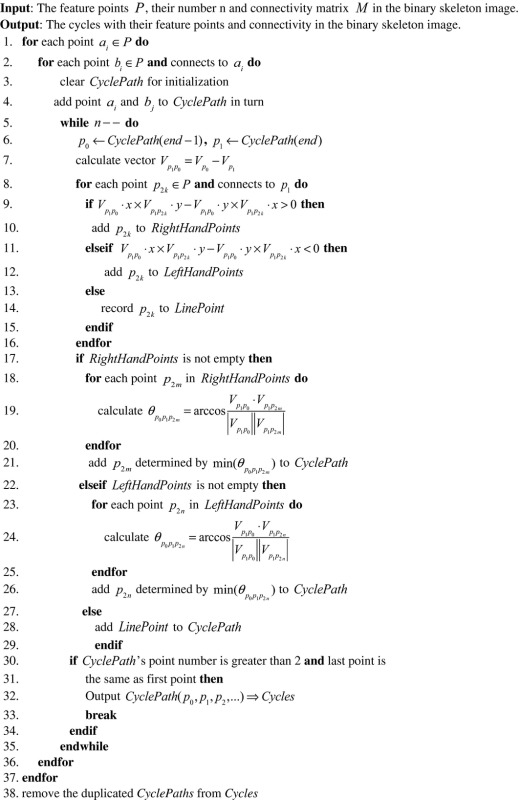

## References

[ece32789-bib-0001] Adams, D. C. , Rohlf, F. J. , & Slice, D. E. (2004). Geometric morphometrics: Ten years of progress following the ‘revolution’. Bolletino di Zoologia, 71(1), 5–16.

[ece32789-bib-0002] Allison, E. H. , & Brand, A. R. (1995). A mark‐recapture experiment on queen scallops, Aequipecten opercularis, on a north Irish sea fishing ground. Journal of the Marine Biological Association of the United Kingdom, 75(2), 323–335.

[ece32789-bib-0003] Berkman, P. A. (1990). The Population Biology of the Antarctic Scallop, *Adamussium colbecki* (Smith 1902) at New Harbor, Ross Sea In Kerry K. R. & Hempel G. (Eds.), Antarctic ecosystems (pp. 281–288). Berlin: Springer.

[ece32789-bib-0004] Bolger, D. T. , Morrison, T. A. , Vance, B. , Lee, D. , & Farid, H. (2012). A computer‐assisted system for photographic mark–recapture analysis. Methods in Ecology & Evolution, 3(5), 813–822.

[ece32789-bib-0005] Bookstein, F. L. (1993). Morphometric tools for landmark data: Geometry and biology. Cambridge: Cambridge University Press.

[ece32789-bib-0006] Chaudhuri, S. , Chatterjee, S. , Katz, N. , Nelson, M. , & Goldbaum, M. (1989). Detection of blood vessels in retinal images using two‐dimensional matched filters. IEEE Transactions on Medical Imaging, 8(3), 263–269. 18230524 10.1109/42.34715

[ece32789-bib-0007] Fearnbach, H. , Durban, J. , Parsons, K. , & Claridge, D. (2012). Photographic mark‐recapture analysis of local dynamics within an open population of dolphins. Ecological Applications, 22(5), 1689–1700. 22908723 10.1890/12-0021.1

[ece32789-bib-0008] Forcada, J. , & Aguilar, A. (2000). Use of photographic identification in capture‐recapture studies of mediterranean monk seals. Marine Mammal Science, 16(4), 767–793.

[ece32789-bib-0009] Gjedrem, T. (1983). Genetic variation in quantitative traits and selective breeding in fish and shellfish. Aquaculture, 33(1), 51–72.

[ece32789-bib-0010] Gonzalez, R. C. , & Woods, R. E. (2007). Digital image processing (3rd ed.). New Jersey: Prentice‐Hall.

[ece32789-bib-0011] Guo, X. , & Luo, Y. (2006). Scallop culture in China. Developments in Aquaculture & Fisheries Science, 35(23), 1143–1161.

[ece32789-bib-0012] Karanth, K. U. , & Nichols, J. D. (1998). Estimation of tiger densities in India using photographic captures and recaptures. Ecology, 79(8), 2852–2862.

[ece32789-bib-0013] Kovács, I. , & Julesz, B. (1993). A closed curve is much more than an incomplete one: Effect of closure in figure‐ground segmentation. Proceedings of the National Academy of Sciences of the United States of America, 90(16), 7495–7497. 8356044 10.1073/pnas.90.16.7495 PMC47168

[ece32789-bib-0014] Langtimm, C. A. , Beck, C. A. , Edwards, H. H. , Fick‐Child, K. J. , Ackerman, B. B. , Barton, S. L. , & Hartley, W. C. (2004). Survival estimates for Florida manatees from the photo‐identification of individuals. Marine Mammal Science, 49(3), 3714–3716.

[ece32789-bib-0015] Mao, J. , Lv, J. , Miao, Y. , Sun, C. , Hu, L. , Zhang, R. , … Bao, Z. (2013). Development of a rapid and efficient method for non‐lethal DNA sampling and genotyping in scallops. PLoS One, 8(7), e68096. 23874509 10.1371/journal.pone.0068096 PMC3706602

[ece32789-bib-0016] Mehlhorn, K. , & Michail, D. (2009). Minimum cycle bases: Faster and simpler. ACM Transactions on Algorithms, 6(1), 5515–5528.

[ece32789-bib-0017] Michellej, L. , & Amandae, B. (2008). Influence of density‐dependent food consumption, foraging and stacking behaviour on the growth rate of the Northern abalone, *Haliotis kamtschatkana* . Aquaculture, 277(2), 24–29.

[ece32789-bib-0018] Michio, S. , & Hiromichi, N. (2013). Mollusk shell structures and their formation mechanism. Canadian Journal of Zoology, 91(91), 529–531.

[ece32789-bib-0019] Reed, J. Z. , Tollit, D. J. , Thompson, P. M. , & Amos, W. (1997). Molecular scatology: The use of molecular genetic analysis to assign species, sex and individual identity to seal faeces. Molecular Ecology, 6(3), 225–234. 9076977 10.1046/j.1365-294x.1997.00175.x

[ece32789-bib-0020] Rohlf, F. J. (1965). Extensions of the Procrustes method for the optimal superimposition of landmarks. Systematic Zoology, 100(39), 283–286.

[ece32789-bib-0021] Rudin, L. I. , Osher, S. , & Fatemi, E. (1992). Nonlinear total variation based noise removal algorithms. Physica D: Nonlinear Phenomena, 60(1), 259–268.

[ece32789-bib-0022] Shumway, S. E. , & Parsons, G. J. (2011). Scallops: Biology, ecology and aquaculture In Short F. T. & Coles R. G. (Eds.), Developments in aquaculture & fisheries science (Vol. 164, pp. 280–281). New York: Elsevier.

[ece32789-bib-0023] Wang, J. (2015). Individual identification from genetic marker data: Developments and accuracy comparisons of methods. Molecular Ecology Resources, 16(1), 352–354. 10.1111/1755-0998.12452 26230747

[ece32789-bib-0024] Wang, Y. , Ji, G. , Lin, P. , & Trucco, E. (2013). Retinal vessel segmentation using multiwavelet kernels and multiscale hierarchical decomposition. Pattern Recognition, 46(8), 2117–2133.

[ece32789-bib-0025] Wang, S. , Meyer, E. , Mckay, J. K. , & Matz, M. V. (2012). 2b‐RAD: A simple and flexible method for genome‐wide genotyping. Nature Methods, 9(8), 808–810. 22609625 10.1038/nmeth.2023

[ece32789-bib-0026] Weiner, S. , & Lowenstam, H. (1989). On biomineralization. London: Oxford University Press.

[ece32789-bib-0027] Williams, B. K. , Nichols, J. D. , & Conroy, M. J. (2002). Analysis and management of animal populations: Modeling estimation, and decision making. New York: Academic Press.

